# A robust PCR for the differentiation of potential virulent strains of *Haemophilus parasuis*

**DOI:** 10.1186/s12917-017-1041-4

**Published:** 2017-05-08

**Authors:** N. Galofré-Milà, F. Correa-Fiz, S. Lacouture, M. Gottschalk, K. Strutzberg-Minder, A. Bensaid, S. Pina-Pedrero, V. Aragon

**Affiliations:** 1 IRTA, Centre de Recerca en Sanitat Animal (CReSA, IRTA-UAB), Campus de la Universitat Autònoma de Barcelona, 08193 Bellaterra, Spain; 2 Faculté de médecine vétérinaire, Université de Montréal, 3200, rue Sicotte, Saint-Hyacinthe, Québec, J2S 2M2 Canada; 3 IVD Innovative Veterinary Diagnostics (IVD GmbH), Albert-Einstein-Str. 5, 30926 Seelze, Germany

**Keywords:** *Haemophilus parasuis*, PCR diagnosis, Bacterial virulence, Glässer’s disease diagnosis

## Abstract

**Background:**

*Haemophilus parasuis* is the etiological agent of Glässer’s disease in swine. *H. parasuis* comprises strains with heterogeneous virulence capacity, from non-virulent to highly virulent. Determination of the pathogenic potential of the strains is important for diagnosis and disease control. The virulence-associated trimeric autotransporters (*vtaA*) genes have been used to predict *H. parasuis* virulence by PCR amplification of their translocator domains. Here, we report a new and improved PCR designed to detect a different domain of the *vtaA* genes, the leader sequence (LS) as a diagnostic tool to predict virulence.

**Methods:**

A collection of 360 ﻿*H. parasuis *﻿strains was tested by PCR with LS specific primers. Results of the PCR were compared with the clinical origin of the strains and, for a subset of strains, with their phagocytosis and serum resistance using a Chi-square test.

**Results:**

LS-PCR was specific to *H. parasuis*, and allowed the differential detection of the leader sequences found in clinical and non-clinical isolates. Significant correlation was observed between the results of the LS-PCR and the clinical origin (organ of isolation) of the strains, as well as with their phagocytosis and serum susceptibility, indicating that this PCR is a good predictor of the virulence of the strains. In addition, this new PCR showed a full correlation with the previously validated PCR based on the translocator domain. LS-PCR could be performed in a wide range of annealing temperatures without losing specificity.

**Conclusion:**

This newly described PCR based on the leader sequence of the *vtaA* genes, LS-PCR, is a robust test for the prediction of the virulence potential of *H. parasuis* strains.

## Background

*Haemophilus parasuis* comprises strains of different virulence, from non-virulent to highly virulent. This bacterial species can be found colonizing the upper respiratory tract of clinically healthy piglets, although some strains can cause invasive systemic disease. Systemic bacterial spreading induces a strong inflammation, which leads to the characteristic lesions of fibrinous polyserositis. The systemic disease caused by *H. parasuis* is known as Glässer’s disease. While *H. parasuis* can be found in basically all farms, disease is developed in a small percentage of them. Several factors are important in disease development, including the presence of other pathogens in the farm and the virulence of the *H. parasuis* strains [1].

Piglets can be colonized by several strains of *H. parasuis*, and therefore, several strains of the bacterium are found in a single farm [2]. This epidemiological situation makes crucial the correct determination of the strain causing the disease outbreak for disease control.

Many studies have been performed in an effort to find virulence markers for *H. parasuis* [3–5]. Some specific genomic sequences were identified by microarray studies to be differentially present in *H. parasuis* strains depending on their clinical origin. Those genes were identified and named virulence associated trimeric autotransporters (*vtaA*) and were classified in three groups based on the *yadA*-like translocator domain sequence [6]. *VtaAs* from groups 1 and 2 were associated to clinical strains [6]. Moreover, two VtaAs from group 1 were demonstrated to play a role in phagocytosis resistance, a virulence mechanism of *H. parasuis* [7, 8]. PCR tests were designed for *H. parasuis* identification and virulence prediction using the *yadA*-like translocator domain of group 1 and 3 *vtaA* [9]. Detection of group 3-translocator was *H. parasuis* species-specific, while detection of group 1-translocator was associated to *H. parasuis* virulent strains [9]. However, the conditions for the proper execution of that PCR were very stringent and complex regarding temperatures and bacterial DNA concentrations, with the risk of losing specificity if not strictly respecting the previously described conditions. Therefore, it was difficult to implement for routine diagnosis. Recently, a genomic comparison of 212 strains confirmed previous reports on the use of *H. parasuis vtaA* for virulence prediction [5]. Two different leader sequences were detected in these genes, and each type was over-represented either in clinical (associated with disease) or non-clinical isolates [5]. Here, we present a new and more robust PCR, based on the leader sequences of the *vtaA* genes.

## Methods

### PCR conditions

Leader sequences of the *vtaA* genes were aligned and compared for primer design, taking into account the clinical origin of the strains (strains isolated from either lesions or nasal cavities of healthy piglets). As forward primer, a common sequence within the leader sequence of *vtaAs* was identified (AAATATTTAGAGTTATTTGGAGTC) and named AV1-F. For specific amplification, two reverse primers were chosen; V1-R (AATATACCTAGTAATACTAGACTTAAAAG), for *vtaA* found in clinical (putative virulent) strains, and NV1-R (CAGAATAAGCAAAATCAGC), for non-clinical nasal (putative non-virulent) strains. Conditions for the PCR reactions were, 1.5 mM MgCl_2_, 0.4 mM each deoxynucleotide triphosphate (dNTP), 400 nM each primer, 1 U GoTaq polymerase (Promega) and 0.3–500 ng of genomic DNA in a final volume of 25 μL in GoTaq buffer (Promega). Cycling conditions were 5 min at 94 °C, followed by 30 cycles of 45 s at 94 °C, 45 s at 52 °C and 1 min at 72 °C, then a final incubation at 72 °C for 7 min.

### Bacterial strains

The PCRs based on the leader sequence of the *vtaA* genes were tested with *H. parasuis* strains previously described [9], together with *H. parasuis* clinical isolates from the diagnostic laboratory services of the Faculty of Veterinary Medicine of the University of Montreal (Canada), the Innovative Veterinary Diagnostics (IVD) Laboratory in Seelze-Letter (Germany) and the laboratory Exopol in Zaragoza (Spain). Nasal isolates were obtained from the nasal cavities of healthy piglets, while the rest of strains were obtained from clinical cases of disease, including systemic, pulmonary and clinical isolates of unknown origin. Association between the PCR results and the clinical origin of the strains was assessed using Pearson’s Chi-square test for categorical data.

Specificity of the leader sequence PCR was confirmed with strains of *Actinobacillus pleuropneumoniae*, *Actinobacillus porcinus*, *Actinobacillus indolicus*, *Actinobacillus minor, Actinobacillus suis, Pasteurella multocida*, *Streptococcus suis* and *Escherichia coli* isolated from swine.

### Virulence assays

The putative virulence of the strains was inferred by the clinical origin of the isolates. For a set of strains phagocytosis and serum resistance assays were performed following previously described protocols [8, 10]. Association between the virulence assays and the PCR results was evaluated using two tailed Fisher exact probability test.

## Results and discussion

**Fig. 1:**
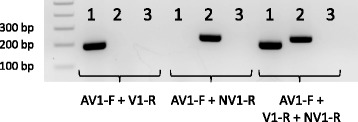
PCR results with the virulent strain Nagasaki (1) and the non-virulent strain F9 (2). Lanes 3 are negative controls. The combination of primers used in each reaction are indicated in the figure

Preliminary assays demonstrated that the reverse primers V1-R (for detection of clinical, putative virulent, strains) and NV1-R (for detection of non-clinical, putative non-virulent, strains) could be used either in pairs with the forward primer AV1-F or in a combination of the three together (Fig. 1), maintaining specificity. The latter multiplex PCR will be referred as LS-PCR for clarity throughout the rest of text.

LS-PCR sensitivity limit was estimated to 0.3 ng of genomic DNA, both for the virulent Nagasaki strain and the non-virulent SW114 strain. Importantly, annealing temperature was modified between 47.5 and 57 °C without losing sensitivity.

A summary of the results obtained with the LS-PCR and the collection of strains is presented in Table 1. To evaluate the association between this new PCR and the potential virulence of *H. parasuis*, results of the LS-PCR and the clinical origin of the strains (nasal versus clinical isolates) were compared using a Chi-Square test. The correlation between the LS-PCR results and the clinical origin (organ of isolation) of the strains was highly significant (Pearson, *p* < 0.0001), indicating that this PCR is a good predictor of the putative virulence of the strains. If the results of the LS-PCR are compared only between the nasal and systemic isolates, the correlation was also highly significant (Pearson, *p* < 0.0001), with 77 out of 120 nasal strains yielding amplification with primer NV1-R and 75 out of 80 systemic strains showing amplification with V1-R.

**Table 1:** Summary of the results of the leader PCR in a collection of 360 strains from different clinical origin

	Number of strains				
PCR results	Total	Isolation site			
		Systemic	Pulmonary	Nasal	Unknown^a^
V	262	75	120	43	24
NV	98	5	14	77	2

^a^clinical isolates, from cases of disease

*V* virulent, *NV* non-virulent

In addition, when the strains previously analyzed using the described PCR for the *yadA* translocator domain of *vtaAs* [9] were tested with the new LS-PCR, a full correlation between both tests was observed. All the strains yielding the amplicon of non-virulent (NV) strains in the LS-PCR were negative in the group 1-*vtaA* PCR, and all the strains that produced the amplicon of clinical virulent (V) strains in the LS-PCR were positive in the group 1-*vtaA* PCR.

To support the results of the LS-PCR, phagocytosis and serum resistance for some strains were contrasted with the PCR results. The association of the LS-PCR results with virulence mechanisms was also significant, although statistically weaker than those found with the clinical origin of the strains, probably due to the lower number of strains used. Table 2 shows the association of the LS-PCR results with serum susceptibility of the strains (Fisher, *p* = 0.04) and with phagocytosis susceptibility (Fisher, *p* = 0.0005). Worth mentioning, two reference strains, serovar 7 strain 174 and serovar 11 strain H465, classified as non-virulent serovars, were identified as virulent by the PCR. In phagocytosis assays, strain 174 was sensitive and strain H465 was resistant to phagocytosis by porcine alveolar macrophages (PAMs). Thus, the results of the LS-PCR and the phagocytosis test were discordant for strain 174. One of the strains that was classified as virulent by this new LS-PCR, strain SL3–2, was sensitive to both phagocytosis and serum. Interestingly, the genome sequence of this strain is available [5] and genome analysis showed the presence of *vtaA*5 and absence of *vtaA* 1, 4, 6, 7, 8 and 9 (from virulent group 1 *vtaAs*) in the genome regions found in other strains. Therefore, strain SL3–2 may be lacking some VtaAs involved in the in vitro virulence assays used in this study, phagocytosis and serum susceptibility, and it may represent a strain with an intermediate phenotype due to reduction in the number of *vtaA*s. On the other hand, five strains isolated from systemic sites (pericardium, brain and abdomen) yielded NV amplification in the LS-PCR. Two of these 5 strains, isolated from brain and pericardium, were studied by serum and phagocytosis susceptibility assays, and were found to be sensitive in both tests (with reduction of bacteria of more than 5 logs after 1 h of incubation with fresh serum, and with more than 50% of PAM with associated bacteria in phagocytosis assays), showing a good correlation between the in vitro tests and the PCR results. It is known that some predisposing factors, such as co-infections [11–13], can facilitate systemic spread of low virulent strains, due to the immune suppression induced by the concomitant pathogens, and this could explain how these serum and phagocytosis sensitive strains were found in systemic organs. In these cases, the main probable cause of disease was not *H. parasuis*, which in fact, was probably acting as a secondary agent. As any other test, this PCR needs to be complemented with clinical, pathological and epidemiological data to reach a meaningful diagnosis of the disease in the farm.

**Table 2:** Association of the PCR results with the serum and phagocytosis susceptibility of selected strains (Fisher, *p* < 0.04 and *p* < 0.0005, respectively); (S: sensible; R: resistant). NV: non-virulent and V: virulent, as determined by the *vtaA* leader sequence PCR

		Serum susceptibility		Phagocytosis susceptibility	
		S	R	S	R
PCR	V	14	16	9	19
	NV	10	2	12	1
		24	18	21	20

**Fig. 2:**
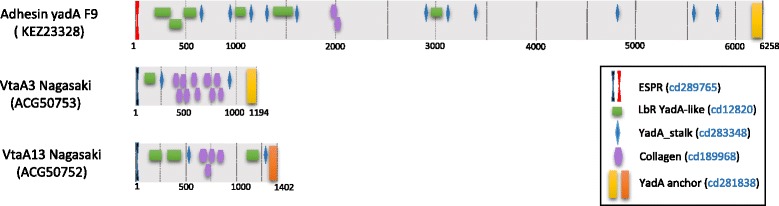
Schematic representation of three *H. parasuis* autotransporters. Each panel shows the domains predicted using CD-search algorithm from NCBI in each protein. At the top, the protein found in non-virulent F9 strain (adhesin YadA) with leader sequence from VtaAs overrepresented in non-clinical isolates (in *red*) and translocator domain from group 1 VtaAs (in *yellow*) is shown. Below, representative proteins from group 1 (VtaA3, ACG50753; group 1 translocator domain indicated in *yellow*) and group 3 (VtaA13, ACG50752; group 3 translocator domain indicated in orange) from Nagasaki strain are shown. The depicted domains are: ESPR (Extended Signal Peptide of Type V secretion system, cd289765), LbR YadA-like (YadA-like, left-handed beta-roll cd12820), YadA stalk (Coiled stalk of trimeric autotransporter adhesion, cd 283,348), Collagen (Collagen triple helix repeat 20 copies, cd189968) and YadA anchor or translocator domain (YadA-like C-terminal region, cd281838). Accession numbers for proteins and domains are shown between brackets. Domains are represented with the same shapes between proteins. Mismatching colors within the same shapes denote low homology between domains from different proteins. Numbers below each protein representation correspond to amino acids

Analysis of the genome of four non-virulent strains (serovar 3 reference strain SW114, serovar 8 reference strain C5, and nasal strains MU21–2 and F9) detected the expected presence of one gene encoding a large trimeric autotransporter with the typical translocator domain of group 3 *vtaA*s, but also a very large trimeric autotransporter of more than 6000 amino acids, with the translocator sequence characteristic of group 1-*vtaA* associated with virulent strains. This latter gene was transcribed under laboratory and biofilm conditions in the F9 strain, indicating that it is a functional gene (GEO:GSE56428). This very large group 1-*vtaA* autotransporter can explain the positive results with the previously described PCR in group 1-*vtaA* for some non-virulent strains, when the conditions of this test were not exactly as described by Olvera et al. [9]. The other domains of this gene, passenger and leader domains, were highly divergent from those found in virulent strains (Fig. 2). In fact, the leader sequence showed high homology to the *vtaAs* found in non-virulent strains. This finding explains the necessity of the stringent conditions for the PCR based on the translocator domain of *vtaAs*, while supporting the use of the LS-PCR for determining the putative virulence of *H. parasuis* strains.

## Conclusion

As conclusion, this newly described PCR based on the leader sequence of the *vtaA* genes, LS-PCR, is a robust test for the prediction of the virulence potential of *H. parasuis* strains.

### Highlights

- Leader sequences of *vtaA* differ depending on the clinical origin of the *H. parasuis* strain
- A robust PCR based on the *vtaA* leader sequence predicts the virulence of *H. parasuis* strains
- This PCR is species-specific and works efficiently at a wide range of annealing temperature

## Abbreviations

dNTP: Deoxynucleotide triphosphate
LS: Leader sequence
NV: Non-virulent
PAM: Porcine alveolar macrophage
V: Virulent
VtaA: Virulence-associated trimeric autotransporters
